# Comparative analysis of *Saccharomyces cerevisiae *WW domains and their interacting proteins

**DOI:** 10.1186/gb-2006-7-4-r30

**Published:** 2006-04-10

**Authors:** Jay R Hesselberth, John P Miller, Anna Golob, Jason E Stajich, Gregory A Michaud, Stanley Fields

**Affiliations:** 1 Department of Genome Sciences, University of Washington, Box 357730, Seattle, WA 98195, USA; 2 Department of Molecular Genetics and Microbiology, Duke University, Durham, NC 27710, USA; 3 Invitrogen, East Main Street, Branford, CT 06405, USA; 4 Department of Medicine, and Howard Hughes Medical Institute, University of Washington, Box 357730, Seattle, WA 98195, USA; 5 Current address: Buck Institute, Redwood Boulevard, Novato, CA 94945, USA

## Abstract

**Background:**

The WW domain is found in a large number of eukaryotic proteins implicated in a variety of cellular processes. WW domains bind proline-rich protein and peptide ligands, but the protein interaction partners of many WW domain-containing proteins in *Saccharomyces cerevisiae *are largely unknown.

**Results:**

We used protein microarray technology to generate a protein interaction map for 12 of the 13 WW domains present in proteins of the yeast *S. cerevisiae*. We observed 587 interactions between these 12 domains and 207 proteins, most of which have not previously been described. We analyzed the representation of functional annotations within the network, identifying enrichments for proteins with peroxisomal localization, as well as for proteins involved in protein turnover and cofactor biosynthesis. We compared orthologs of the interacting proteins to identify conserved motifs known to mediate WW domain interactions, and found substantial evidence for the structural conservation of such binding motifs throughout the yeast lineages. The comparative approach also revealed that several of the WW domain-containing proteins themselves have evolutionarily conserved WW domain binding sites, suggesting a functional role for inter- or intramolecular association between proteins that harbor WW domains. On the basis of these results, we propose a model for the tuning of interactions between WW domains and their protein interaction partners.

**Conclusion:**

Protein microarrays provide an appealing alternative to existing techniques for the construction of protein interaction networks. Here we built a network composed of WW domain-protein interactions that illuminates novel features of WW domain-containing proteins and their protein interaction partners.

## Abstract 2

A protein interaction map for 12 of the 13 WW domains present in the proteins of *S. cerevisiae *was generated by using protein microarray data.

## Background

### Methods for building protein interaction networks

The assembly of networks of interacting proteins and genes has provided a new perspective on the organization and regulation of cellular processes, allowing the superimposition and interpretation of a variety of types of functional information [1]. Detailed analysis of these networks has revealed underlying hierarchies of interactions ('network motifs') [2], which illustrate the common topologies adopted by groups of interacting genes and proteins. To date, protein interaction networks built from experimental data have been based on either high-throughput versions of the yeast two-hybrid (Y2H) assay [3,4], or protein epitope-tag affinity purification/mass spectrometry (AP-MS) [5,6]. The methods are complementary: Y2H identifies binary protein-protein interactions whereas AP-MS establishes the members of co-purifying protein complexes. Both methods will likely be required to accurately model local topologies within large networks [7], and they have been used to interconnect thousands of proteins.

However, both of these approaches have inherent drawbacks. They each suffer from their own classes of false positives: for example, self-activating protein fusions can lead to artifactual Y2H results, and high abundance proteins can contaminate protein pulldowns in the AP-MS strategy. Conversely, false negatives occur in each method due to their respective constraints. The Y2H assay demands that the interacting proteins be functional in the context of a fusion and that interactions occur in the nucleus to be detected; for this reason, many proteins (for example, membrane proteins) are not amenable to the standard assay. The AP-MS approach can miss transiently interacting proteins, proteins that do not stay associated during purification, and complexes not soluble through the procedure. In addition, AP-MS approaches demand that the epitope tag not affect a protein's proper folding and inclusion within a complex. Because of these technical drawbacks, protein interaction maps are both incomplete and contain interactions that are not biologically relevant.

Recently, a third experimental approach, protein microarrays, has been developed that circumvents some of these problems. In this approach, purified proteins are presented in a format for *in vitro *binding studies, providing a platform for a variety of protein interaction experiments (for example, lipid-protein, small molecule-protein and protein-protein interactions [8]). The protein microarrays have certain advantages: they are comprehensive, encompassing for yeast the great majority of proteins, including proteins of low cellular abundance; they are rapid to screen and analyze; and they likely contain proteins that exhibit native post-translational modifications when the normal host is used as the source of protein. An additional feature is that array experiments are performed under a uniform set of conditions, thus replacing the disparate cellular milieus found *in vivo *with a single set of experimental parameters *in vitro*. The arrays also have limitations: some proteins cannot be expressed and purified; co-purifying proteins may be present on the array; and the modification of array probes (for example, biotinylation) may influence their binding properties.

### Classification of WW domains in yeast

The WW domain is a well-characterized, highly conserved protein domain found in multiple, disparate proteins and subcellular contexts in a number of organisms [9,10], including humans, in which the dysfunction of these proteins may contribute to multiple disease states [11]. The domain adopts a compact, globular fold with three β-sheets, forming two grooves that serve as sites for ligand binding [12]. WW domains bind proline-rich peptide or protein ligands [11]; this ligand recognition is mediated by sets of conserved residues within the domain [13,14], as observed in structures of WW domains in complex with peptide ligands [15,16]. Based on the presence of signature residues, a classification scheme has been proposed for WW domains [13,14]. WW domains within these classifications have particular ligand specificities: group I domains bind Pro-Pro-Xaa-Tyr (PY) motifs [11,14]; group II/III domains bind poly-proline motifs [13]; and group IV domains bind proline motifs containing phosphorylated serine or threonine residues [14].

**Figure 1:**
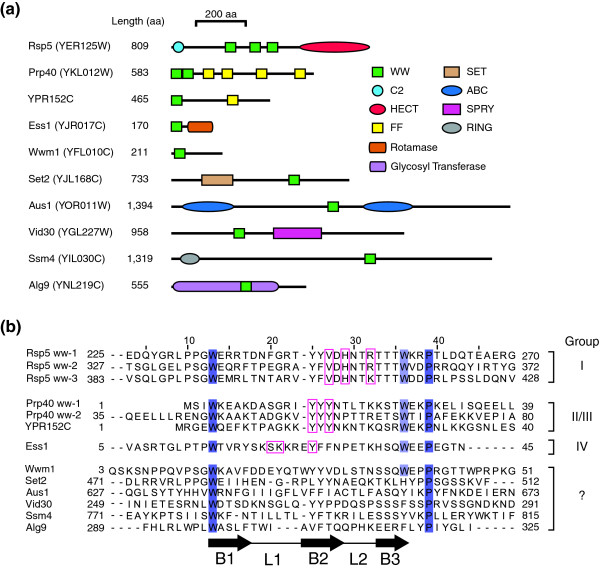
Motifs in yeast WW domain proteins and WW sequence alignment. **(a) **Ten yeast proteins contain a total of thirteen WW domains. **(b) **Multiple sequence alignment of the 13 WW domains. The domains from Rsp5 and Prp40 are named corresponding to their occurrence from amino to carboxyl terminus. Conservation of the tryptophan residue at position 13 and the proline residue at position 39, as well as partial conservation of the tryptophan at position 36 define the WW domain (filled blue boxes). The sequences shown were purified as fusions to either MBP or GST. Residues boxed in red residues indicate the sequence determinants that put the WW domains into three different classes: groups I, II/III and IV [13]. Six of the WW domains do not conform to any of the classifications.

Ten proteins from *Saccharomyces cerevisiae *contain 13 WW domains (Rsp5 contains three WW domains; Prp40 contains two WW domains) (Figure 1a). The domains are defined by conserved residues at particular positions (for example, tryptophan at positions 13 and 36; proline at position 39), but overall very little of the WW domain sequence is conserved (Figure 1b). Several of these proteins have been well characterized. Rsp5 (YER125W) is a ubiquitin ligase that participates in a variety of cellular processes, including vesicle sorting and protein modification within the endoplasmic reticulum (ER) [17]. Ssm4 (YIL030C) is another ubiquitin ligase that associates with the ER and functions in Matα 2 repressor degradation [18,19]. The histone methyltransferase Set2 (YJL168C) and the peptidyl-prolyl isomerase Ess1 (YJR017C) interact with the carboxy-terminal domain of RNA Pol II via its phosphorylated Ser-Pro motifs [20,21] and participate in the regulation of transcription at the level of chromatin modification (Set2) and polymerase remodeling (Ess1). Prp40 (YKL012W) participates in mRNA splicing, interacting with Msl5 and Mud2 during the splicing reaction, and it has also been linked to the Pol II machinery [22].

Five of the *S. cerevisiae *WW domains are derived from proteins about which little is known. These WW domains do not conform to the canonical groupings of WW domains (Figure 1b), and thus the interaction specificities of these domains cannot be predicted. Vid30 (YGL227W) has a putative role in the vacuolar catabolite degradation of fructose-1,6-bisphosphatase [23]. Alg9 (YNL219C) is an ER-associated protein involved in glycoprotein biosynthesis [24]; its human homolog is associated with congenital disorders of glycosylation [25]. Wwm1 (YFL010C) has been implicated in yeast apoptosis, and interacts genetically with Mca1, the meta-caspase that initiates the peroxide-induced apoptotic response in yeast [26,27]. Aus1 (YOR011W) is involved in the uptake of sterols [28]. The YPR152C protein is listed only as a 'hypothetical protein' by the *Saccharomyces* Genome Database [29], and has no functional annotation.

The three WW domains from Rsp5 belong to the group I class; the two WW domains from Prp40 and the domain from Ypr152c belong to the group II/III class; and the domain from Ess1 belongs to the group IV class. The WW domains from Prp40 [22] and Ess1 [30] interact with phosphorylated Ser/Thr-Pro motifs, though further characterization via NMR indicates that the Prp40 domains also bind peptide ligands containing PY and PPΨΨP motifs [15]. The remaining six WW domains from Set2, Ssm4, Aus1, Vid30, Alg9 and Wwm1 do not conform to any of the known classifications, possibly indicating a specialization of these domains with concomitant changes in structure and ligand specificity. Except for the domain present in Wwm1, these meta-WW domains lack the conserved tryptophan residue at position 36 in the domain (Figure 1b), in addition to residues used for the group classification scheme.

## Results and discussion

### Identification of yeast WW domain-protein interactions

We used protein microarrays to generate a protein interaction map of yeast WW domain-containing proteins. The microarrays were constructed by printing 4,088 proteins from *S. cerevisiae *in duplicate on nitrocellulose-coated glass slides. Other proteins printed on the arrays served as controls, including biotinylated antibodies for the detection of the biotinylated probes and gluthathione S-transferase for the analysis of binding specificity. In Y2H experiments with several of these WW domains present in DNA-binding domain fusions as either full-length proteins or isolated domains, we were unsuccessful in recovering previously reported interactions and unable to test many of the constructs due to their transcriptional self-activation (data not shown). Therefore, protein microarrays provided an alternative method to identify the protein interaction partners of these domains.

**Figure 2:**
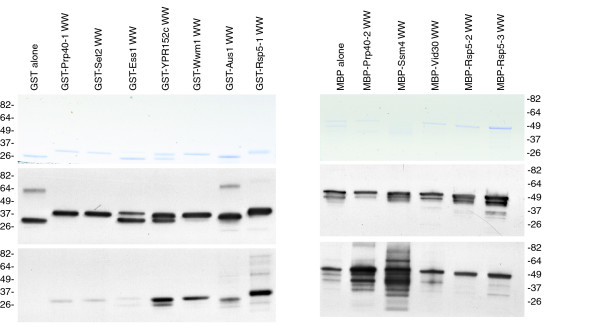
Purification of WW domain fusion proteins. Coomassie-stained SDS-PAGE gel of WW domain fusion proteins following protein purification (top panels), western blot detection of fusion protein expression with anti-GST antibody (left middle panel) or anti-myc antibody (right middle panel), and biotinylation of fusion proteins observed by binding of HRP-conjugated streptavidin (bottom panels) are shown.

**Figure 3:**
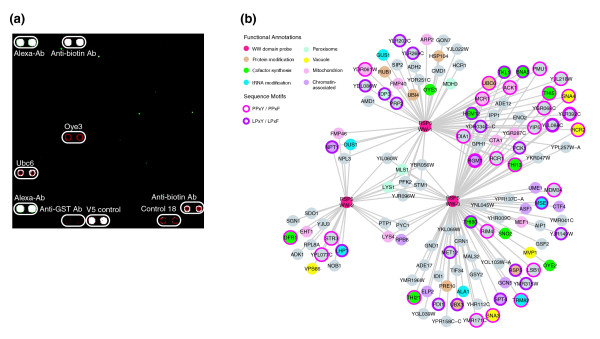
Protein microarray data and the Rsp5 network. **(a) **A microarray was probed with the first WW domain from Rsp5 and interactions were visualized via application of dye-labeled streptavidin and fluorescent scanning. Following data processing, two proteins (Ubc6 and Oye3) had signals above background. Control proteins (dye-labeled and biotinylated proteins) are indicated. **(b) **Interactions involving the WW domains from Rsp5. A total of 124 proteins were identified using the WW domains from Rsp5. Functional annotations are superimposed on the network using filled circles and outlines.

We expressed each of the individual domains in *Escherichia coli *as a fusion to either glutathione S-transferase (GST) or maltose binding protein, and purified the fusion proteins (Figure 2). During purification, WW domain fusion proteins were biotinylated using an amine-reactive biotinylation reagent, and each of the purified domains was used to probe duplicate protein microarrays. We were unable to obtain sufficient expression of either type of fusion protein containing the WW domain from Alg9, and thus focused on the remaining 12 WW domain probes. Protein-protein interactions on the microarrays were detected by the addition of fluorophore-conjugated streptavidin, and individual spots on the microarray were visualized by fluorescence scanning (Figure 3a). Previously, protein-protein and protein-lipid interactions identified using protein microarrays were shown to be highly reproducible [31]. However, because of the importance of reproducibility in any protein interaction experiment, we applied each probe protein to two separate microarrays. After data processing, only those proteins found as high-confidence interactions were selected for further analysis. We defined high-confidence interactions to be those in which four independent observations of the interaction were made (that is, signals greater than three standard deviations above the mean spot fluorescence for a protein printed in duplicate on two separate microarrays). To identify interactions that might be platform-specific, we compared our initial data to a set of 13 supplementary protein microarray experiments that had previously been carried out (GAM, unpublished data). We removed 15 proteins from our data set that were found in more than half of these experiments, leaving 587 high-confidence interactions between 12 WW domains and 207 proteins (Additional data file 1).

### Properties of the WW domain network

**Figure 4:**
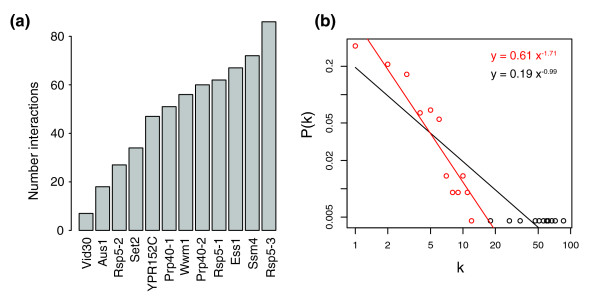
WW domain network properties. **(a) **The number of interaction partners identified using each WW domain probe. **(b) **Log-log plot of the node degree distribution within the WW domain network. Black circles represent WW domain probes and red circles represent protein interactors; power law fits to data sets including (black line) and excluding (red line) WW domain probe are shown.

Within this network, the number of interactions observed with different WW domain probes varied from 86 interactions for the third WW domain of Rsp5 to 7 for Vid30 (Figure 4a); a recent study of a human 14-3-3 protein using protein microarrays identified 20 proteins as 14-3-3 interactors [32]. The three domains from Rsp5 together interacted with 124 proteins (about 60% of the network), 45 of which were identified solely by these domains (Figure 3b). Conversely, the first domain from Prp40 interacted with one protein uniquely and the domain from Set2 had no unique partners. In general, there is a large degree of overlap within the network, as 53 proteins were found by at least 4 different domain probes.

We used the Gene Ontology (GO) hierarchy [33] to identify regions of the network that are enriched for particular classifications. The network was first split into 12 subnetworks, each consisting of a single WW domain probe and its interaction partners. These subnetworks contain a number of significant (*P *< 0.05 using a hypergeometric test) enrichments of GO annotations (Additional data file 2). In particular, an enrichment of proteins involved in cofactor metabolism suggests a role for Rsp5 in the assembly or localization of the biosynthetic enzymes responsible for the metabolism of thiamine and other cofactors (Figure 3b). Enrichment of proteins within the network that localize to the peroxisome suggests that Rsp5, Ssm4 and Prp40 may be involved in processes within this organelle. Proteins containing WW domains also affect the localization and degradation of several proteins from the ER and other membranous intracellular compartments. For example, deletion of Ssm4 abrogates degradation of the ER transmembrane protein Ubc6 [18], and Rsp5-mediated ubiquitination of plasma membrane proteins directs their internalization and targeting to the endosomal-lysosomal pathway [17]. In addition, we observe interactions with several other ER proteins (for example, Rsp5 interacts with Ubc6 and Pdi1) and GTP-hydrolyzing proteins involved in vesicle transport (for example, Ssm4 interacts with Ypt6 and Ess1 interacts with Ypt53).

Protein-protein interaction networks have a common underlying topology in which the distribution of node degrees can be fit to a power law [34]. Intuitively, this observation is consistent with protein functions: many proteins are specialized and interact with relatively few partners, whereas relatively few proteins are involved in numerous processes and interact with many partners. However, discrepancies can arise when this analysis is applied to small, sampled subsets of larger networks [35]. Our interaction network differs from existing networks because it is focused on a single type of protein domain, and is likely, therefore, to be more heavily sampled (that is, more locally complete) than previous large-scale screens. The node degree distribution of the WW domain network exhibits the expected 'scale-free' topology of protein interaction networks (Figure 4b).

**Figure 5:**
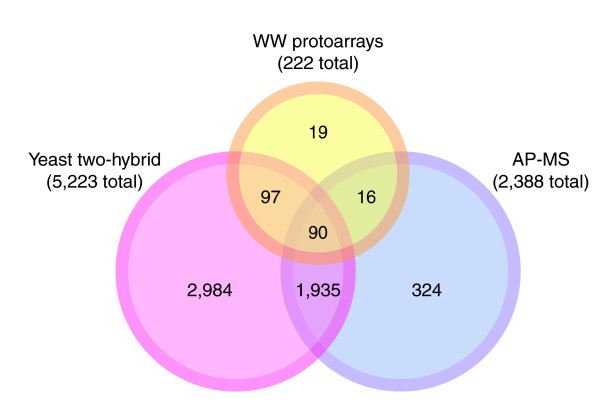
Venn diagram illustrating the representation of yeast proteins involved in protein-protein interactions found using yeast two-hybrid (Y2H) assay, protein epitope-tag affinity purification/mass spectrometry (AP-MS) and protein microarray strategies.

We searched the network for groups of proteins having conserved protein domains from the eMotif database [36], but found no significantly enriched protein domains except for the WW domain itself (data not shown). This observation is consistent with the fact that binding sites recognized by WW domains are short primary sequences as opposed to sizable protein domains. We also used data compiled for Y2H and AP-MS experiments available from the *Saccharomyces* Genome Database [29] to identify 19 proteins within the network that have not been reported as having known protein interaction partners (Figure 5). Analysis of these proteins using the GO Term Finder available from the *Saccharomyces* Genome Database indicates no consistent functional annotation within this set of proteins.

Within the interaction network generated in this study, a total of 13 interactions have support from experimental studies, bioinformatic approaches, or both. Eight interactions have been observed previously by either the Y2H assay [3] or AP-MS [5]. Five of these involved the ubiquitin ligase Rsp5, which targets multiple proteins for degradation [37], two involve interactions with Prp40, and the final one is the interaction between Ess1 and Bcy1, a regulatory subunit of cAMP-dependent protein kinase A [5]. Two interactions involving Rsp5 were found in a recent screen for Rsp5 substrates [38]. A probabilistic network of functional linkages [1] supports eight interactions that we identified (Additional data file 3). We searched for orthologous interactions ('interologs' [39]) between our dataset and the recently generated protein interaction maps of *Drosophila melanogaster *[40], *Caenorhabditis elegans *[41] and *Homo sapiens *[42] but found no conserved interactions.

Given the low degree of overlap between these protein microarray data and existing datasets, validation of these interactions by other approaches is an important step prior to further analysis of the biology of these interactions. For example, a reversed microarray experiment could be used to address array-based artifacts, in which microarrays would be assembled using the WW domain-fusion proteins as array features, and these arrays would be probed with the interacting proteins that were originally identified. Alternatively, epitope-tagged versions of the WW domains could be introduced into cells, and interacting proteins would be identified using immunoprecipitation and western blotting or affinity purification and mass spectrometry; a similar strategy was used to identify proteins that interact with human WW domain-containing proteins [43].

### WW ligand sequence motif representation

To address ligand specificity, we compiled a list of primary sequence motifs of known WW domain-ligands from the literature and searched the proteins in our network for occurrences of these motifs. Within the network, 28 proteins have canonical PY motifs and 5 have poly-proline motifs. Twenty-six proteins have PPR motifs, and 38 proteins have a degenerate PY motif, the LPxY motif, which was previously shown to be a determinant for Rsp5 specificity [44]; 24 of these 38 interacted with Rsp5 (Figure 3b). Twenty proteins have more than one motif or possess motifs from multiple classes (Additional data file 4). We found a significant enrichment of proteins with PY and LPxY motifs (*P* < 10^-8 ^and 0.02, respectively, using a binomial test) relative to all proteins present on the microarrays. In the *S. cerevisiae *proteome, approximately 250 proteins contain PY motifs (4% of all proteins) and 400 proteins contain LPxY motifs (7%). In contrast, approximately 30% of the proteins in the WW domain network contain either PY or LPxY motifs.

The prevalence of the PY motif within the network is expected given the group I classification of the three WW domains from Rsp5. Of the 124 proteins that interacted with these domains, 27 have PY motifs (Figure 3b); only 9 proteins in the network have a PY motif and did not interact with a WW domain from Rsp5. Consistent with its role as an E3 ubiquitin protein ligase, Rsp5 interacted with several proteins involved in protein modification and turnover, including members of the ubiquitin modification system (for example, Ubi4, Ubc6 and Ubp10), and ubiquitin-like modifications (Rub1). In addition, we observed the known self-interaction between the third WW domain of Rsp5 and the Rsp5 protein on the microarray [45]. Surprisingly, we did not observe interactions between the Rsp5 WW domain probes and two members of a known Rsp5 complex, Bul1 and Bul2 [46], both of which are present on our arrays and contain PY motifs. As these proteins are members of a complex, it is possible that accessory proteins needed to mediate the interaction of Rsp5 with Bul1 and Bul2 are not present on the microarray.

A total of 8 proteins in the network have matches to the poly-proline motifs (PPLP and PPPP), and 26 proteins have matches to the PPR motif. Several of these proteins are promiscuous; for example, 2 proteins with poly-proline motifs and 6 proteins with PPR motifs interacted with half or more of the WW domains. This scattered distribution may reflect some intrinsic property of interactions between these ligand classes and WW domains, such as relatively weak affinities between these molecules in the context of microarrays.

The WW domain from Ess1 belongs to the group IV class, which binds phosphorylated ligands. However, because we do not know the phosphorylation states of proteins on the microarrays, we cannot assess the proportion of phosphorylation-dependent interactions within the network. Rpo21, the Pol II subunit containing the carboxy-terminal domain that is bound by Ess1 when phosphorylated, is not present on the microarrays. However, proteins containing WW domains have been proposed to mediate a physical coupling between the transcription and splicing processes in yeast [10]. Consistent with this association, we observed an interaction between Ess1 and Prp2, a DEAD-box RNA-dependent ATPase required for the first step of mRNA splicing [47].

Approximately 43% of the proteins within the network have matches to the canonical ligand motifs known to mediate WW domain interactions. The absence of known motifs in other interacting proteins could be due to any of several reasons. First, isolated WW domains may recognize novel sequence motifs when they are removed from their protein context. Second, they may bind to structural motifs that have yet to be identified at a primary sequence level. Third, other accessory proteins may be needed for WW-containing proteins to recognize their targets.

The lack of known motifs could also be due to more general consequences of using the microarray strategy to identify protein ligands. In a microarray experiment, the concentration of probe protein defines the upper limit of affinity for an interaction. Our probes were applied at low micromolar concentrations, and, therefore, interactions with K_D _values higher than this limit would be missed; most of the K_D _values measured for WW domain:ligand interactions are in the 10 to 100 μM range [13]. On the other hand, the concentration of probe may be so high as to recover interactions that are not physiologically relevant. These false-positives could account for spurious interactions with proteins that lack canonical ligand motifs, or have a particular motif but are not bound *in vivo*.

As nearly half of the proteins in the network do not have recognizable WW domain ligand motifs, we searched for novel motifs within the network using motif identification software, including MEME [48] and a network-based motif sampler [49]. These approaches did not identify any novel motifs, indicating either that most common motifs have been identified, or that additional parameters such as structural information may be needed to define novel motifs. However, the MEME searches converged on degenerate versions of the PY and LPxY motifs. Many WW domains possess some level of recognition flexibility toward peptide ligands *in vitro*, and we asked whether this same versatility was reflected among the proteins within the WW domain network.

### Phylogenetic evidence for structural conservation of WW domain ligands

We used a comparative genomics approach to analyze the distribution and conservation of WW domain binding sites. Similar approaches have been used to annotate genomes, to search for conserved functional DNA elements, such as transcription factor binding sites [50,51], to discover novel protein interactions [52], and to delineate receptor-ligand interactions [53]. Recently, the strategy was used to analyze the yeast SH3 domain interaction network, illustrating that the comparative approach, in combination with protein disorder prediction, was effective in recovering known interactions and predicting novel ones [54]. Because the peptide ligands bound by WW domains are small, well-defined and sufficient for binding (for example, Pro-Pro-Xaa-Tyr), the search for evolutionarily conserved WW binding sites within protein partners can potentially be reduced to the identification of conserved stretches of amino acid residues.

**Figure 6:**
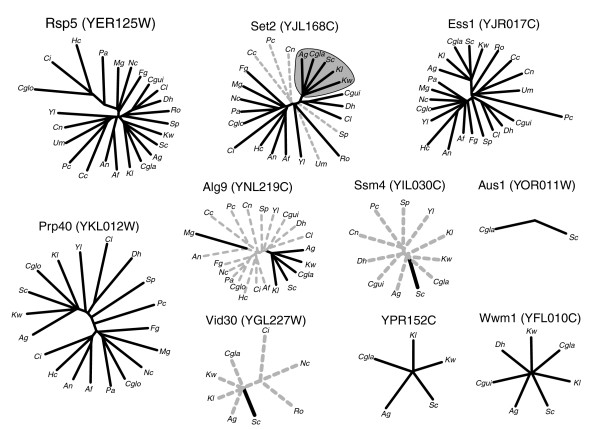
Phylogenetic conservation of WW domains among yeast lineages. Radial trees were generated based upon multiple alignments for orthologs culled from 24 yeast species. Solid lines indicate lineages in which the WW domain is maintained in the orthologous proteins, whereas dashed lines indicate those proteins in which the WW domain is not present. In the Set2 ortholog group, the WW domains highlighted in gray are most similar to the meta-WW domain in *S. cerevisiae*, whereas in the other lineages the WW domain conforms to the group II/III classification. Organism abbreviations are *Saccharomyces cerevisiae *(*Sc*),*Candida guilliermondii *(*Cgui*),*Candida glabrata *(*Cgla*),*Chaetomium globosum *(*Cglo*),*Kluyveromyces waltii *(*Kw*),*Kluyveromyces lactis *(*Kl*),*Yarrowia lipolytica *(*Yl*),*Candida lusitaniae *(*Cl*),*Debaryomyces hansenii *(*Dh*),*Schizosaccharomyces pombe *(*Sp*),*Pneumocystis carinii *(*Pc*),*Fusarium graminearum *(*Fg*),*Magnaporthe grisea *(*Mg*),*Neurospora crassa *(*Nc*),*Podospora anserina *(*Pa*),*Aspergillus fumigatus *(*Af*),*Aspergillus nidulans *(*An*),*Ashbya gosypii *(*Ag*),*Histoplasma capsulatum *(*Hc*),*Coccidioides immitis *(*Ci*), *Ustilago maydis *(*Um*),*Cryptococcus neoformans *(*Cn*),*Coprinus cinereus *(*Cc*),*and Rhizopus oryzae *(*Ro*).

We compiled genomic sequences for several yeast species in the *ascomycete *and *basidomycete *lineages and searched for orthologs of proteins in our interaction network using the best-hit reciprocal BLAST method [55]. Of the 207 *S. cerevisiae *proteins in the network, 191 have at least one ortholog among the 24 yeast species analyzed. We also analyzed the conservation of the WW domains themselves among yeast lineages (Figure 6). The WW domains in Rsp5, Prp40, Ess1, Wwm1, Aus1 and Ypr152c are maintained in all the yeast species. The WW domain in Set2 orthologs is either missing, or is found as one of two classes: the group II/III domain, or, in species closely related to *S. cerevisiae*, a meta-WW domain, which lacks the residues defining the group II/III class. The distribution of WW domains among Alg9 orthologs is mainly restricted to species closely related to *S. cerevisiae*, whereas that of Ssm4 and Vid30 is only in the *S. cerevisiae *lineage.

**Figure 7:**
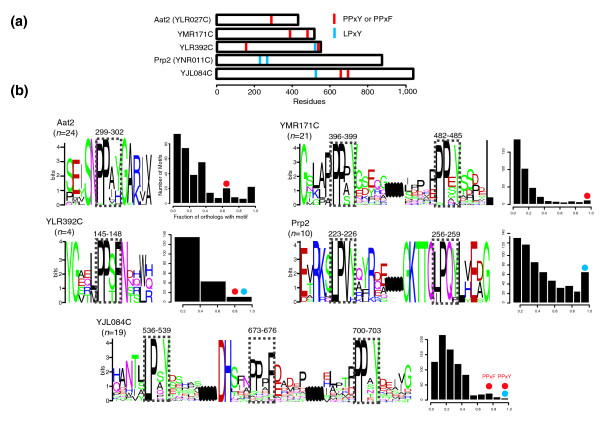
Phylogenetic conservation of the WW ligand motifs within yeast proteins. **(a) **Positions of primary sequence motifs within *S. cerevisiae *Aat2, Ymr171c, Ylr392c, Prp2, and Yjl084c. **(b) **Logo representations [68] of the conserved region within the set of orthologs. The number of orthologs in each set is indicated. Gray dashed boxes highlight the conserved motifs; numbers indicate the position of the motif within the *S. cerevisiae *protein. Histograms represent the level of conservation of all *S. cerevisiae *X-X-x-X sequence determinants within the set of orthologs. Colored circles mark the bins that contain the PPxY, PPxF and LPxY motifs.

These sets of orthologous protein sequences were used to generate multiple sequence alignments, which were examined for the conservation of known primary sequence motifs. In several instances, known WW ligand sequence motifs are conserved among the lineage of interactor orthologs (Figure 7; Additional data file 4). Moreover, we found evidence suggesting that WW domains have sufficient recognition malleability to bind structurally similar peptide ligands within the PY (PPxY) and LPxY ligand classes. Both the PPxY and LPxY motifs were found in sets of orthologs as: an invariant sequence; multiple sequences in which the 'x' position varies; or multiple sequences in which the tyrosine is replaced with structurally similar residues (predominantly phenylalanine but in some instances histidine or tryptophan). Although the first two classes were expected, the third class has not been previously observed in a biological context. However, the group I WW domains exhibit recognition flexibility *in vitro*. Previously, the specificity of the Yap65 WW domain was assessed using an array of peptides encompassing each single alanine substitution of the peptide ligand, demonstrating that phenylalanine is a functional replacement for tyrosine within the PPxY motif [56]. Several group I WW domains also exhibit this recognition flexibility [57]; the structure of a Nedd4 WW domain-PPxY ligand indicated that peptide binding uses a groove that recognizes the N-substituted Pro-Pro sequence, forming a large pocket that accommodates the tyrosyl side chain [16]. It is possible that phenylalanine side chains are accommodated by this pocket, and that the subtle tyrosine to phenylalanine structural change may be used in biological contexts for the tuning of WW domain-ligand interactions.

We analyzed several conserved motifs in detail (Figure 7). Ymr171c, an endosomal protein of unknown function that interacted with the third WW domain from Rsp5, harbors two PPxY motifs that are maintained in nearly all of its 21 orthologs. Aat2 is an aspartate aminotransferase that localizes to peroxisomes during oleate utilization [58]. It contains a single PPxY motif that is maintained as PPxH and PPxF in several of the orthologs. Ylr392c contains single instances of the PPxY, PPxF and LPxY motifs, each of which is conserved among its three orthologs. Ylr392c interacted with the first and third WW domains of Rsp5, a finding that is supported by its prior identification via AP-MS as a member of an Rsp5 complex [5]. Yjl084c contains instances of the PPxY, PPxF and LPxY motifs. The PPxY and LPxY motifs are maintained in all 19 orthologs, while the PPxF motif is present in 15 of the orthologs. Yjl084c interacted with the first and third domains of Rsp5, and is known to be phosphorylated by Cdk1 [59]. Finally, Prp2 is an essential RNA helicase that participates in the early steps of mRNA splicing. Prp2 has two LPxY motifs that are conserved among its ten orthologs. Prp2 was found by five WW domain probes, possibly indicating a reduction in specificity for the LPxY motif.

**Figure 8:**
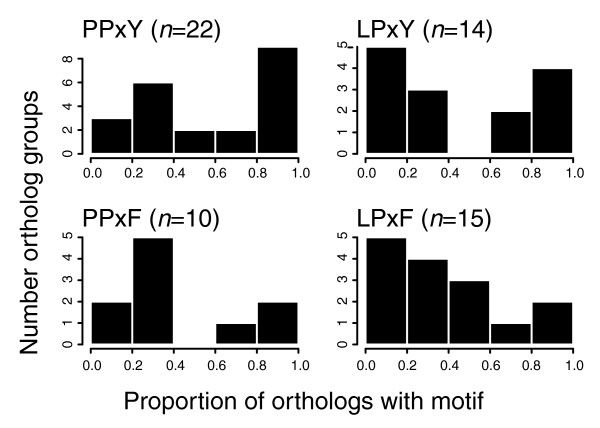
Histograms representing the levels of conservation for the PPxY, PPxF, LPxY and LPxF motifs among 54 orthologous protein sets.

These motifs may represent structural determinants that are evolutionarily maintained because of a selective pressure applied by their interactions with WW domain-containing proteins. This hypothesis relies on the assumption that the presence of a protein sequence motif (for example, PPxY) is sufficient to mediate an interaction with a WW domain. We tested this assumption by asking whether these putative WW domain recognition determinants are more conserved than similar determinants. For each set of orthologs, we used the *S. cerevisiae *protein as a reference point and asked to what extent other determinants of a similar form are conserved. For example, both the PPxY and LPxY motifs can be generalized as tripeptides with an intervening residue (that is, X-X-x-X). For each such tripeptide in the *S. cerevisiae *protein, we determined the proportion of orthologs that maintained the three residues, allowing all substitutions at the 'x' position. We generated histograms of these data, and labeled the bins that contain the putative determinant (for example, PPxY) present in the *S. cerevisiae *protein (Figure 7b). In each case except that of Aat2, the putative determinants are among the most highly conserved motifs within the set of orthologs, suggesting that these sequences are being actively maintained. In the Aat2 lineage, PPxY is found as PPxH and PPxF in several of the orthologs, reducing its apparent conservation level. Of the 54 ortholog groups that have instances of the PPxY, PPxF, LPxY or LPxF motifs, we found 27 orthologous protein sets in which the motif is maintained in more than half of the orthologs, suggesting that maintenance of these determinants is common among the proteins found to interact with WW domains (Figure 8).

When structural malleability within WW domain ligands was observed, the results were initially disregarded as *in vitro *artifacts. Here, we have presented evidence that recognition versatility is sufficiently widespread as to be conserved in several protein lineages from evolutionarily distant yeast species. To address the limits of this conservation, we performed a re-evaluation of a recent study [43] of human WW domain interactions based on epitope tagging and AP-MS. Several of the co-purifying proteins do not have matches to the canonical sequence motifs that were initially analyzed [43]. However, we found that many of the human proteins have matches to the PPxF and LPxY motifs, including splicing and transcription factors (for example, PPxF and LPxY in U2AF2, LPxY in CPSF1) (Additional data file 5).

### Several WW domain proteins have conserved WW domain binding sites

**Figure 9:**
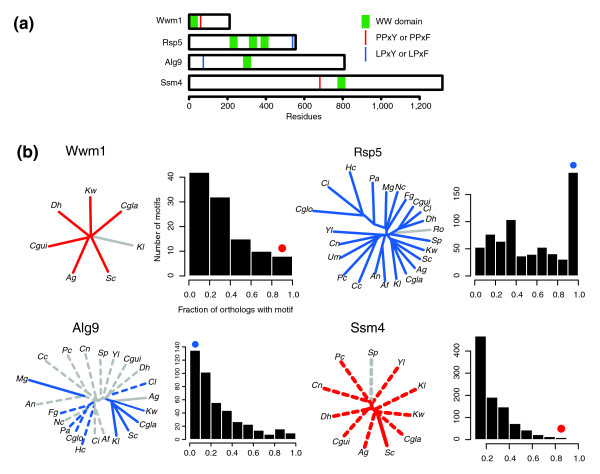
Co-occurrence of WW domains and WW domain binding sites. **(a) **Positions of the WW domains (green bars) and conserved primary sequence motifs (PPxY/F in red, LPxY/F in blue) in Wwm1, Rsp5, Alg9 and Ssm4. **(b) **Radial trees and motif conservation for the ortholog groups of each protein. Organism abbreviations are *Saccharomyces cerevisiae *(*Sc*),*Candida guilliermondii *(*Cgui*),*Candida glabrata *(*Cgla*),*Chaetomium globosum *(*Cglo*),*Kluyveromyces waltii *(*Kw*),*Kluyveromyces lactis *(*Kl*),*Yarrowia lipolytica *(*Yl*),*Candida lusitaniae *(*Cl*),*Debaryomyces hansenii *(*Dh*),*Schizosaccharomyces pombe *(*Sp*),*Pneumocystis carinii *(*Pc*),*Fusarium graminearum *(*Fg*),*Magnaporthe grisea *(*Mg*),*Neurospora crassa *(*Nc*),*Podospora anserina *(*Pa*),*Aspergillus fumigatus *(*Af*),*Aspergillus nidulans *(*An*),*Ashbya gosypii *(*Ag*),*Histoplasma capsulatum *(*Hc*),*Coccidioides immitis *(*Ci*), *Ustilago maydis *(*Um*),*Cryptococcus neoformans *(*Cn*),*Coprinus cinereus *(*Cc*),*and Rhizopus oryzae *(*Ro*). Solid branches indicate lineages in which the WW domain is present; dashed lines indicate the absence of a WW domain. Colored branches indicate lineage in which a motif is present; lineages in gray lack the motif. The histograms represent the relative conservation of *S. cerevisiae *motifs of the form X-X-x-X among the orthologs. Red and blue dots indicate the bins that contain the highlighted motifs.

Searches for primary sequence motifs within the WW domain-interacting orthologs indicated that several of the WW domain-containing proteins themselves have evolutionarily conserved WW domain binding sites (Figure 9a). A similar observation [60] was made for Rsp5, which binds peptides harboring the LPxY motif that is found at the carboxyl terminus of Rsp5. Our analysis revealed that Alg9 also has a conserved LPxY motif that in some lineages is coincident with presence of the WW domain, possibly indicating a co-evolving domain and binding site (Figure 9b). In addition, the Wwm1 and Ssm4 proteins harbor PY motifs (PPxY in Wwm1, PPxF in Ssm4), which are maintained in nearly all of their respective orthologs. We analyzed these proteins for the conservation of *S. cerevisiae *protein motifs and found that for Rsp5, Wwm1 and Ssm4, the putative WW domain binding sites are among the most conserved motifs within these proteins (Figure 9b). The LPxY determinant in Alg9 is less well-conserved, which may indicate that it is not used as a WW domain recognition site.

The pattern of conservation for the Wwm1, Rsp5 and Ssm4 proteins is suggestive of two separate types of evolutionary maintenance. The first is self-interaction, as when the WW domains and recognition sites are co-maintained in Wwm1 and Rsp5. We observed an interaction between the third WW domain of Rsp5 and the Rsp5 protein on the microarray, supporting the conservation a WW domain binding site. In our study, Wwm1 was present on the microarrays but did not interact with the Wwm1 WW domain probe. The second type of maintenance is binding of a conserved recognition site to another WW domain-containing protein that is present throughout the lineage. For Ssm4, the WW domain is present only in the *S. cerevisiae *protein, whereas WW domain binding sites are present in nearly all of the orthologs. As both Ssm4 and Rsp5 are ubiquitin ligases, it is possible that the conserved PPxF site in Ssm4 mediates an interaction with Rsp5; the presence of the WW domain in the *S. cerevisiae *Ssm4 ortholog may thus reflect a unique functional specialization. In our study, Ssm4 was not present on the microarrays.

The role of Wwm1 in the yeast apoptotic response [27] may be mediated by its interaction with either itself or other proteins containing WW domains, possibly serving to propagate some signal necessary for regulation of this response. Wwm1 interacted with Pai3, the cytoplasmic inhibitor of yeast saccharopepsin [61]. As the apoptotic cascade in higher eukaryotes is initiated by a series of proteolytic cleavage events, the Wwm1-Pai3 interaction may point to a similar protease-initiated cascade of signaling events in yeast.

### A model for WW domain interaction evolution

**Figure 10:**
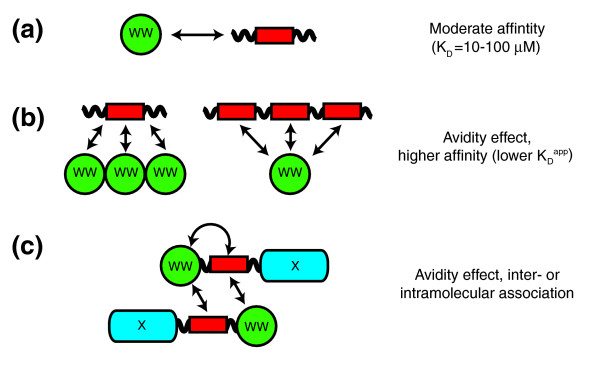
A model for the optimization of interactions between WW domains and protein ligands. WW domains are colored green, WW ligand binding motifs are colored red, and auxiliary protein domains are in blue.

Isolated WW domains bind their cognate ligands weakly *in vitro*, with K_D_s in the 10 to 100 μM range [13,14] (Figure 10a). However, the biological context of many WW domains and their protein ligands likely serves to increase the affinity of these interactions. Two broad classes of binding modes could increase the apparent affinity of interactions (Figure 10b). One class is represented by proteins that have multiple WW domains and bind ligands with isolated motifs, whereas the other class contains proteins with a single WW domain whose ligands contain multiple binding sites. Both of these situations are frequently observed: Rsp5 and Prp40 in *S. cerevisiae *and several human proteins contain multiple WW domains [43]. Conversely, the *S. cerevisiae *Ess1 protein (Pin1 in humans) interacts with several repeats of the phospho-Ser/Thr-Pro motif in the Pol II carboxy-terminal domain [10]. Coincident WW domain and WW binding sites (Figure 10c) such as those found in the Rsp5, Ssm4, Alg9 and Wwm1 proteins could influence function by serving as sites for either intra- or intermolecular association. Such associations could provide a mechanism for self-imposed regulation, or could play a more active role by increasing the local concentration of an ancillary functional domain, labeled 'X' in Figure 10c. Analysis of the interactions of the WW domains of human Nedd4 family proteins showed that whereas some proteins were recognized uniquely by a WW domain, others were recognized by multiple WW domains [43], supporting a model for interaction specificity tuning. WW domains may thus act as scaffolds in the construction of multi-protein complexes by providing a mechanism for the optimization of specificity and affinity for the interactions between WW domains and their protein partners.

## Conclusion

We have constructed a network of yeast WW domain interactions using protein microarrays, the first such domain-specific network built using this strategy. Protein microarray technology is sufficiently orthogonal to existing techniques to allow the recovery of a number of previously unobserved, but biologically relevant protein interactions, and will be useful in the future for refining and expanding protein interaction maps. A comparative genomic approach uncovered evidence for a previously unappreciated level of structural malleability in the conservation of WW domain ligands. The comparative approach also revealed that WW domain-containing proteins often themselves contain conserved WW domain binding sites, indicating a role for multimerization in WW domain protein function. WW domains have been shown to possess recognition flexibility *in vitro*, and this versatility manifests itself *in vivo *on an evolutionary scale.

## Materials and methods

### WW fusion protein construction and purification

The sequence for each of the 13 WW domains including approximately 10 amino acids amino-terminal to the first tryptophan and approximately 10 amino acids carboxy-terminal to the conserved proline residue were cloned into *E. coli *expression vectors pMAL-c2x (New England Biolabs, Beverly, MA, USA) or pGEX-4T (Amersham Biosciences, Uppsala, Sweden) to generate maltose binding protein or GST fusions, respectively. A 300 ml culture of Luria-Bertani broth (LB) was inoculated with a starter culture of WW-domain fusion-containing bacteria, induced to express protein with isopropyl-beta-D-thiogalactopyranoside (IPTG), and harvested as described in the manufacturer's protocol. Supernatants from sonicated cell lysates were passed over equilibrated amylose resin (New England Biolabs) or glutathione-beads (Amersham Biosciences). Proteins were biotinylated by the addition of 50 μg/ml NHS-LC-LC-biotin (Pierce Biotechnology, Rockford, IL, USA) to the columns, washed with phosphate-buffered saline (PBS), and eluted with either 10 mM maltose or 20 mM glutathione. Proteins were assessed for expression and purity by coomassie staining and western blot against the fusion protein, and for biotinylation by detection with horseradish peroxidase (HRP)-conjugated streptavidin (Figure 2). Concentration was assessed by comparison to known amounts of proteins on SDS-PAGE gels, as well as by comparison to protein standards in Bradford assays and absorbance at 280 nm.

### Yeast proteome collection

The yeast proteome collection was derived from the yeast clone collection of 5,800 yeast open reading frames [31]. The identity of each clone was verified using 5' end sequencing. Expression of GST-tagged protein by each clone was tested using western blotting and detection with an anti-GST antibody. The 4,088 clones that passed both quality control measures were purified using high-throughput affinity chromatography as previously described [31].

### Yeast protein microarray manufacturing

Commercially available protein microarrays were manufactured by Invitrogen (Carlsbad, CA, USA). A contact-type printer (Omnigrid, Genomic Solutions, Ann Arbor, MI, USA) equipped with 48 matched quill-type pins was used to deposit each of 4,088 purified yeast proteins along with a set of control elements in duplicate spots on 1" × 3" nitrocellulose-coated glass slides. The printing of these arrays was carried out in a cold room under dust-free conditions to preserve the integrity of both samples and printed microarrays. Each lot of slides was subjected to a quality control procedure that included a gross visual inspection of all the printed slides for imperfections. The second step consisted of a more detailed characterization of each spot on the array. Since each of the proteins was tagged with GST, this quality control procedure was accomplished by using an antibody detection protocol specific for GST. This procedure measures the variability in spot morphology, the number of missing spots, the presence of control spots, and the amount of protein deposited in each spot. The number of missing spots on the arrays was less than 1%, and the median spot size was 130 mm.

### Protein array probing and data analysis

Microarray experiments were carried out in a cold room (4°C) as described by the manufacturer (Invitrogen). Briefly, arrays were probed with 300 μl of a solution containing 50 μM biotinylated probe protein in PBS on ice in horizontally positioned Atlas glass hybridization chambers for 90 minutes. Following incubation, the arrays were washed 3 times with 2 ml of PBS, followed by the addition of 2 ml of PBS containing a 400 μg/ml of Alexa Fluor 647-streptavidin. The arrays were incubated for 30 minutes and then washed three times with 2 ml of PBS, removed from the incubation chamber and air-dried by hand-shaking the slides. Fluorescent scans of each protein microarray were obtained using an Axon GenePix scanner (Molecular Devices, Sunnyvale, CA, USA) and were manually processed. The protein microarrays are printed onto a total of 48 blocks, which are separable based on their coordinates. Because of local variations in the background on each array, we analyzed each block separately. Counts from each yeast protein spot (excluding control proteins) within a block were combined and a trimmed mean (removing the top and bottom 10% of the data) was calculated. Spots with counts greater than three standard deviations above this trimmed mean were selected as positives. A protein scored as an initial positive is one that was found in duplicate in two independent array experiments. Protein microarray data generated in this study have been deposited at the NCBI Gene Expression Omnibus [62] under accession GSE3758.

To identify false-positive and platform-specific interactions, we compared our data set to 13 interaction data sets previously collected using the yeast protein microarrays (GAM, unpublished data). Proteins in our dataset that appeared in more than half (seven or more) of these supplemental data sets were removed. We also examined the interactions to identify proteins that were specific to the MBP or GST fusions, as these could represent false-positives arising from interaction with maltose binding protein (MBP) or GST, but did not find any fusion-specific interactions.

### Primary sequence motif analysis

Sequences for yeast strains were compiled from the Resource for Fungal Comparative Genomics [63], which compiles and annotates fungal genomic sequences generated by multiple sources. Orthologs of *S. cerevisiae *proteins were identified from 24 yeast species in the *ascomycetes *and *basidomycetes *lineages using the reciprocal BLAST method [55]. In addition to best reciprocal matches, we required that at least 80% of the sequence was aligned. The organisms used in the analysis were *S. cerevisiae*, *Candida guilliermondii*, *Candida globrata*, *Chaetomium globosum*, *Kluyveromyces waltii*, *Kluyveromyces lactis*, *Yarrowia lipolytica*, *Candida lusitaniae*, *Debaryomyces hansenii*, *Schizosaccharomyces pombe*, *Pneumocystis carinii*, *Fusarium graminearum*, *Magnaporthe grisea*, *Neurospora crassa*, *Podospora anserina*, *Aspergillus fumigatus*, *Aspergillus nidulans*, *Ashbya gosypii*, *Histoplasma capsulatum*, *Coccidioides immitis*, *Ustilago maydis*, *Cryptococcus neoformans*, *Coprinus cinereus*, *and Rhizopus oryzae*. Multiple alignments were generated with T-Coffee using default values [64] and visualized using Jalview [65]. Phylogenetic trees were generated using the Phylip software package [66].

### Protein interaction network analysis

The protein network was searched for groups of enriched GO classifications using the GO classification resource available through the *Saccharomyces* Genome Database [29], and eMotif classifications obtained from the *Saccharomyces* Genome Database were used to search for protein domain enrichment within the network. Network visualization was done using Cytoscape [67].

## Additional data files

The following additional data are available with the online version of this paper. Additional data file 1 is a compilation of WW domain-protein interactions recovered using the protein microarray strategy. The first column corresponds to the name of the WW domain probe, and the second column is the systematic name of its protein interaction partner. Additional data file 2 shows enrichment of GO classifications within the WW domain network. Additional data file 3 shows the overlap between the WW domain interaction data set and previously generated protein-protein interaction networks. Additional data file 4 is the primary sequence motif representation in the network. Motifs were identified using regular expressions (poly-proline 'P{4,}', PY 'PP\w [YF]', LPxY 'LP\w [YF]', PPR 'PP [RK]'). Additional data file 5 lists the primary sequence motifs from the human WW domain interaction data set. Motifs that were previously assessed are highlighted by blue headers.

## Supplementary Material

Additional data file 1 The first column corresponds to the name of the WW domain probe, and the second column is the systematic name of its protein interaction partner. Click here for file

Additional data file 2 Enrichment of GO classifications within the WW domain network. Click here for file

Additional data file 3 Overlap between the WW domain interaction data set and previously generated protein-protein interaction networks. Click here for file

Additional data file 4 Motifs were identified using regular expressions (poly-proline 'P{4,}', PY 'PP\w [YF]', LPxY 'LP\w [YF]', PPR 'PP [RK]'). Click here for file

Additional data file 5 Motifs that were previously assessed are highlighted by blue headers. Click here for file
